# Public health nurses for case finding, assessment and referral of community-dwelling socially isolated and/or lonely older adults

**DOI:** 10.3389/fpubh.2023.1114792

**Published:** 2023-03-22

**Authors:** Jonathan Howland, Amanda Stone

**Affiliations:** ^1^Department of Emergency Medicine, Boston University Chobanian & Avedisian School of Medicine, Boston, MA, United States; ^2^Massachusets Association of Public Health Nurses, Milton, MA, United States

**Keywords:** social isolation, loneliness, older adults, public health nurses, aging population, social determinants of health

## Introduction

Social isolation and/or loneliness among community-dwelling older adults are measurable (1, 2) and emerging issues receiving increasing global attention from public health professionals, healthcare providers, policy makers, and researchers. Loneliness is recognized as a social determinant of health (3). The World Health Organization identifies both social isolation and loneliness as important but neglected Social Determinants of Health in the older adult population. Three metrics qualify social isolation and/or loneliness as public health problems with serious implications: association with excess morbidity and mortality; high prevalence; and increasing prevalence. Evidence suggests that effective mitigation is feasible. Strategies for reducing older adult social isolation and/or loneliness should include the expertise of community-based Public Health Nurses (PHNs).

### Morbidity and mortality

There is compelling evidence that social isolation and loneliness are independent risks for morbidity and mortality equivalent to behavioral risks, such as smoking, obesity, physical deconditioning, and substance use disorder (4). A meta-analysis by Holt-Lunstad et al. of 70 studies including 3,407,134 participants that examined the relationship between social isolation, loneliness, living alone and all-cause mortality, found that across studies, social isolation increased mortality risk by 29%, loneliness by 26% and living alone by 32% (4). While some health problems can cause social isolation and loneliness, these investigators included only prospective studies (average follow-up 7 years) and excluded subjects with severe or terminal disease, thereby providing support for the hypothesis that the lack of social connectedness plays a causal role in premature mortality (4). A similar meta-analysis of 35 prospective studies with over 77,000 participants found that loneliness increased risk for all cause mortality by 22%, independent of depression (5). Other studies have found significant associations between measures of social connectedness and risk for coronary artery disease and stroke (6, 7) and hypertension (8), dementia and cognitive decline (9), mental health issues (10), including anxiety and depression (11), chronic diseases, including elevated cholesterol levels, diabetes, and self-reported poor health (12), suicidal ideation and suicide (13–16), elder abuse (17), diminished overall quality of life (18, 19), and damaging health-related behaviors, including smoking and other substance use, poor nutrition and decreased physical activity (20–23). Moreover, studies indicate that social connectedness is associated with health recovery and reduced healthcare utilization. A study of British patients with heart failure found that compared with non-lonely heart failure patients, those who reported loneliness were at 68% increased risk of hospitalization, 57% increased risk of emergency department visits, and 26% increased risk for outpatient visits (24). Related studies found that patients with limited social support who had been discharged from hospital following myocardial infarction had increased mortality, readmissions, and subsequent infarctions (25, 26).

### Prevalence

Social isolation and loneliness are prevalent. The National Health and Aging Trends Study estimates that 24% of independently-living US older adults (≥65 years) are socially isolated (27). A study by Perissinotto et al. found that 43% of those 60 years of age or older report loneliness (28). Another study that used data from the National Social Life, Health, and Aging Project found that 19% of older adults (≥60 years) reported frequent loneliness and 29% reported occasional loneliness (29).

While social isolation and loneliness often occur together and are frequently conflated, they are distinct conditions. Social isolation is measured by the frequency and quality of social contacts, whereas loneliness is a subjective state, a feeling, which can occur in the presence or absence of social isolation. Nonetheless, the health effects of social isolation, loneliness, and living alone appear equivalent in association with all-cause mortality (4).

### Trends

In the US, every day an estimated 10,000 people turn 65 years of age and become eligible for Medicare (30). The fastest growing age group is those aged ≥85 years (31). Such growth in these age cohorts is unprecedented in US history. The combination of a rapidly aging population and increasing proportions of older adults that experience social isolation and/or loneliness will inevitably increase the numbers of older adults at risk for the consequences of social disconnection. Although there are limited data on trends in the prevalence of social isolation and loneliness, it is estimated that by 2050 the older adult US population will approach 90 million (32). Assuming that the current prevalence estimate (24%) (27) holds constant, there would be as many as 21.6 million socially isolated older adults by that year. Putnam provides extensive documentation of the decline of civic and social engagement in the US during the second half of the 20th century (33). He describes widespread decreases, relative to the 1950's, in membership in religious groups, labor unions, parent-teacher associations, League of Women Voters, veterans' groups, fraternal organizations, and other group social activities (33). He argues that America is losing the social capital that comes from people interacting in-person in groups of mutual interest. Repeat questions in the 1985 and 2004 General Social Survey found that the number of adults in the US who reported having no one with whom to discuss important matters nearly tripled (34).

These trends have cost implications that will further challenge US health and social entitlement programs. Although at this point, the literature on the economic burdens of social isolation and/or loneliness are scarce, an exception is a recent (2020) review by Mihalopoulos, et al. (35). This review identified four papers reporting on excess healthcare costs associated with actual or perceived social disengagement, of which two focused on loneliness, one on social isolation, and one on both social isolation and loneliness. All the studies on social isolation found associations with excess healthcare costs and two of the three studies on loneliness found association with higher healthcare costs (35).

### Intervention

While nascent, there is evidence that social isolation and loneliness can be mitigated, although, overall, the current evidence base comprises many studies that are compromised by small sample size, lack of theoretical framework, and weak study designs (36). Nonetheless, increasing awareness of the prevalence and consequences of older adult social isolation and loneliness will inevitably lead to a proliferation of policy innovations and more rigorous intervention trials, nationally and globally. England and Japan have already established ministries of loneliness; several countries, including England, Denmark, Australia, New Zealand, and the Netherlands have initiated regional and national programs to promote social connectedness among their older adult populations (37). Although presently there is a paucity of rigorous evaluation research on interventions for social isolation and loneliness, there are exceptions. A recent meta-analysis of interventions associated with reductions in social isolation and/or loneliness found that many of the 44 studies included had limited effects, but both animal therapy and videoconferencing significantly reduced loneliness in long-term care (38). A systematic review found that effective interventions include social activity and support (39). Some well-designed randomized trials have shown promising results for low-cost programs such a periodic phone calls delivered by trained laypersons. In a randomized trial of periodic empathetic phone calls by trained laypersons, older adults receiving calls showed significant reductions in loneliness, depression, generalized anxiety, and mental health status, relative to controls (40).

Pursuant to the public health model, interventions could target primary, secondary, and/or tertiary prevention. As noted in a recent consensus statement (36), primary prevention could focus on designing physical environments that create opportunities for social interaction during routine activities of daily life (36). Secondary prevention could involve outreach and screening for individuals who have recently retired, lost a spouse, or are otherwise at risk for loneliness and/or social isolation (36). Tertiary prevention could engage those identified as experiencing chronic loneliness and/or social isolation in counseling and programs that promote social interaction and networking (36).

## Discussion

To address social isolation and/or loneliness among community-dwelling older adults a public health approach is needed (36, 41) to collect and analyze data, define the scope of the problem at the community level, identify risk and protective factors and implement and evaluate intervention programs. As with other public health problems, effective intervention for older adult social isolation and loneliness requires case finding of individuals with unmet health and social service needs, assessment to understand which health and social services are needed, and referral to appropriate health and social services providers.

The American Public Health Association defines public health nursing as, “the practice of promoting and protecting the health of populations using knowledge from nursing, social, and public health sciences” (42). Public health nursing practice includes: the design, implementation and evaluation of public health programs and interventions; advocacy; community engagement; coalition building; health education; and policy development and reform. The essential role of the PHN is to promote, protect and improve population health. With an emphasis on primary prevention and a focus on vulnerable and underserved populations, PHNs work to address root causes and the multiple determinants of health to create the conditions in which all people can achieve optimal health and well-being. PHNs develop partnerships to strengthen connections and build capacity between healthcare systems, public health organizations and community networks. They engage and collaborate with health professionals, community members and key stakeholders across sectors to leverage public health priorities and advocate for policies, programs, and resources, and culturally appropriate services aimed at preventing disease, promoting health and advancing health equity. Thus, PHNs have the necessary skills and are in a unique position to conduct outreach, case finding, assessment and referral of socially isolated, lonely and underserved community residents. Mobilizing multisectoral partnerships further strengthens the safety net for this vulnerable population and enhances prevention efforts and community integration.

A recent National Academy of Sciences (NAS) consensus report underscores the potential role of the health care system in addressing older adult social isolation and loneliness (27). This report emphasizes the role of primary care providers in intervening with their socially isolated and lonely patients (27). Primary care providers may encounter socially isolated and/or lonely older adults in their practice and undoubtably have a role to play. Nonetheless, they may not have the awareness, skills or time to identify socially disconnected patients and the NAS report (27) and others (43) have noted that many socially disconnected older adults who could benefit from intervention remain hidden from health and social service providers. Whereas, the linkage between medical and social service providers is traditionally weak (27), this relationship is typically strong among PHNs.

Social service providers, first responders, neighbors, or relatives may know of socially isolated or lonely older adults in their communities, but this knowledge may not translate to intervention due to lack of assessment or referral expertise. As part of their practice, PHNs routinely interface with a broad array of civic workers and community residents who can provide information on socially isolated or lonely older adults for follow-up case finding by PHNs. As community-based interventions that address older adult social disconnectedness proliferate, PHNs are valued assets for developing, implementing and evaluating strategies to protect the health and well-being of this at-risk population.

Case-finding, assessment and referral must be essential components of programs designed to address social isolation and loneliness in community-dwelling older adults. Community PHNs have local knowledge, and established relationships and are well positioned to play a critical role in addressing social disconnection among older adults. PHNs practice surveillance for reportable health problems as part of their routine activities. As healthcare providers they have the formal tools and informal skills to assess undiagnosed and unmanaged health problems, mental health distress, indicators of dementia, substance abuse, malnutrition, and physical, financial, or emotional abuse.

Authors of a recent review of methods for identifying older people at risk of social isolation and loneliness concluded that it is unclear as to which methods, or combination of methods, are most successful (44). More research is needed to assess the cost-effectiveness of various case finding techniques. It is notable, however, that a report on this topic by the British Hidden Citizens, A Campaign to End Loneliness, underscored the need for an identification tool to be used by frontline personnel who provide support to older people (43). We concur and argue that existing community-based providers, including public health nurses, have much to contribute to locating, assessing and referring socially isolated and/or lonely residents from within the communities in which they serve.

## Author contributions

JH and AS contributed equally to the conceptualization and writing of this article. Both authors approved the submitted and revised versions.
